# First report of *pfhrp2* and *pfhrp3* gene deletions compromising HRP2-based malaria rapid diagnostic tests in Malawi

**DOI:** 10.1186/s40249-025-01368-8

**Published:** 2025-10-09

**Authors:** Johnsy Mary Louis, Ernest Mazigo, Hojong Jun, Wang-Jong Lee, Jadidan Hada Syahada, Fadhila Fitriana, Fauzi Muh, Wanjoo Chun, Won Sun Park, Se Jin Lee, Sunghun Na, Feng Lu, Eun-Teak Han, Jin-Hee Han

**Affiliations:** 1 Present Address: Department of Medical Environmental Biology and Tropical Medicine, School of Medicine, Kangwon National University, Chuncheon, Republic of Korea; 2 Present Address: Department of Parasitic Diseases, National Institute for Medical Research, Dar Es Salaam, Tanzania; 3 Department of Epidemiology and Tropical Diseases, Faculty of Public Health, Universitas Diponegoro, Semarang, Indonesia; 4 Department of Pharmacology, School of Medicine, Kangwon National University, Chuncheon, Republic of Korea; 5 Department of Physiology, School of Medicine, Kangwon National University, Chuncheon, Republic of Korea; 6 Department of Obstetrics and Gynecology, Kangwon National University Hospital, Chuncheon, Republic of Korea; 7 Department of Pathogen Biology and Immunology, School of Medicine, Yangzhou University, Yangzhou, China; 8 Institute of Medical Sciences, Kangwon National University, Chuncheon, Republic of Korea

**Keywords:** *Plasmodium falciparum*, *pfhrp2*, *pfhrp3*, Gene deletion, Diagnostic accuracy, Rapid diagnostic test

## Abstract

**Background:**

Histidine Rich Protein 2-based rapid diagnostic tests (HRP2-based RDTs) are widely used for malaria diagnosis in Malawi, but their accuracy may be compromised by *Plasmodium falciparum* parasites lacking the *P. falciparum histidine rich protein 2* (*pfhrp2*) and *P. falciparum histidine rich protein 3* (*pfhrp3*) genes. While such deletions have been reported in other malaria-endemic countries, their presence and diagnostic impact in Malawi remain unknown. This study aimed to determine the prevalence of *pfhrp2/pfhrp3* gene deletions in Malawi and their effect on the diagnostic accuracy of HRP2-based RDTs relative to light microscopy and qPCR.

**Methods:**

A cross-sectional study was conducted between December 2020 and June 2021, enrolling 1582 participants from referral hospitals in Mzuzu (*n* = 1186) and Lilongwe (*n* = 396). Malaria diagnosis was performed using RDTs, microscopy, and qPCR. A total of 391 *P. falciparum* positive samples were analyzed for *pfhrp2/pfhrp3* gene deletions using multiplex qPCR. Diagnostic accuracy metrics, such as sensitivity and specificity, were calculated with 95% confidence intervals. Spearman correlation was applied to assess associations involving log-transformed parasitemia, unpaired *t*-tests were used to compare diagnostic methods, and Mann–Whitney tests were used to compare symptomatic and asymptomatic groups.

**Results:**

Malaria prevalence was higher in Lilongwe (45.2%) than in Mzuzu (22.9%). Infections in Lilongwe were predominantly asymptomatic (94.2%), whereas Mzuzu had mostly symptomatic cases (97.1%) (*P* < 0.0002). RDTs demonstrated higher sensitivity of 78.5% (95% *CI*: 74.6–82.1%) than microscopy 64.8% (95% *CI*: 60.3–69.1), but slightly lower specificity, with 93.6% (95% *CI*: 92.0–95.0%) for RDT compared to 95.4% (95% *CI*: 94.0–96.6%) for microscopy. Dual *pfhrp2/3* gene deletions were found in 24 (15.0%) isolates from Lilongwe and 24 (10.4%) from Mzuzu. All dual-deleted samples were false negative by RDT but were positive by microscopy and qPCR.

**Conclusions:**

This study is the first to report *pfhrp2/3* gene deletions in Malawi. The presence of these deletions may compromise the performance of HRP2-based RDTs, indicating the need to reassess diagnostic strategies in affected regions.

**Supplementary Information:**

The online version contains supplementary material available at 10.1186/s40249-025-01368-8.

## Background

Malaria is still considered a significant public health concern [1]. In 2023, there were an estimated 263 million cases globally, 94% (247 million) of them occurring in sub-Saharan African countries including Malawi. More than 95% of Malawi population live in malaria endemic areas [2]. In 2021, Malawi accounted for about 8% of all malaria cases in Eastern and Southern African countries [3]. In 2023, Malawi contributed to 1.8% of the global malaria cases, 1.2% of global deaths, according to the most recent World Malaria Report [2]. *Plasmodium falciparum* was responsible for more than 95% of malaria infections and deaths in Malawi [3].

Since 2000, a dramatic decrease in global malaria cases and deaths has been reported in sub-Saharan African countries [4]. The decrease resulted from the scaling-up of interventions including the use of malaria rapid diagnostic tests (RDTs) for detection of malaria infections, which was recommended by the WHO in the early 1990s. Since its deployment, RDTs have considerably facilitated early malaria diagnosis procedures, especially in rural malaria endemic regions and other areas where good microscopy is not feasible. For instance, of 3.1 billion RDTs that were sold between 2010 and 2020, 81% were used in sub-Saharan countries, making it the primary malaria diagnostic technique [3]. However, although the use of RDTs is increasing, the global rise in the prevalence of *P. falciparum* isolates lacking the *histidine-rich protein 2* (*pfhrp2*) and histidine-rich protein 3 (*pfhrp3*) genes is compromising the diagnostic effectiveness of *Pf*HRP2-based tests [5]. In sub-Saharan Africa, *pfhrp2* and *pfhrp3* deletions have been increasingly reported in countries such as Equatorial Guinea [6], Mozambique [7], Eritrea [8] with some regions demonstrating deletion rates exceeding 5% [5, 9]. Based on these findings, national malaria control programs in countries like Eritrea and Uganda have updated their diagnostic policies, shifting from HRP2-based RDTs to alternative diagnostic tools, such as parasite lactate dehydrogenase (pLDH)-based tests [10, 11].

Accurate diagnosis of malaria is a fundamental strategy for its control [3]. RDTs detect *Plasmodium* specific antigens, primarily either PfHRP2 or parasite-specific lactate dehydrogenase (pLDH) antigen, from a drop of blood in malaria positive patients [12]. The kits specific for *P. falciparum* are designed to capture histidine-rich protein 2 (PfHRP2), which is encoded by the *pfhrp2* gene located in the sub-telomeric region of chromosome 8 [13]. The *pfhrp2* gene shares 85–90% sequence homology in nucleotide-flanking repeats with *pfhrp3*, which causes them to cross-react in RDTs [13]. As RDTs detect an inert protein component of *P. falciparum* parasites, detection methods using PfHRP2/PfHRP3 are thus limited as proxy measures of parasite density. The sensitivity of RDTs falls sharply when parasite density drops below 100 to 500 parasites/µl [14, 15]. Moreover, the persistence of PfHRP2/PfHRP3 in the bloodstream for several weeks after parasite clearance has been a matter of concern regarding the accuracy of RDT [16].

Based on the WHO guidelines, each country should establish the status and surveillance of gene deletion trends over time [16]. Re-assessment of the national diagnostic strategy has to be done when more than 5% of *P. falciparum* clinical infections are missed by HRP2-based RDTs [17]. This study aimed to investigate the prevalence of *P. falciparum* and *pfhrp2/3* gene deletions in isolates from Mzuzu and Lilongwe, Malawi. Additionally, it assessed how *pfhrp2/3* gene deletions and parasite density impact the accuracy, sensitivity, and specificity of RDTs and microscopy. Importantly, this is the first study to report the presence of *pfhrp2* and *pfhrp3* gene deletions in Malawi. The identification of *P. falciparum* parasites lacking these genes, along with their detection patterns using field diagnostic tools, provides valuable evidence of the effectiveness of the current malaria surveillance and diagnostic strategies in the country.

## Methods

### Study site, population, and sampling

**Fig. 1:**
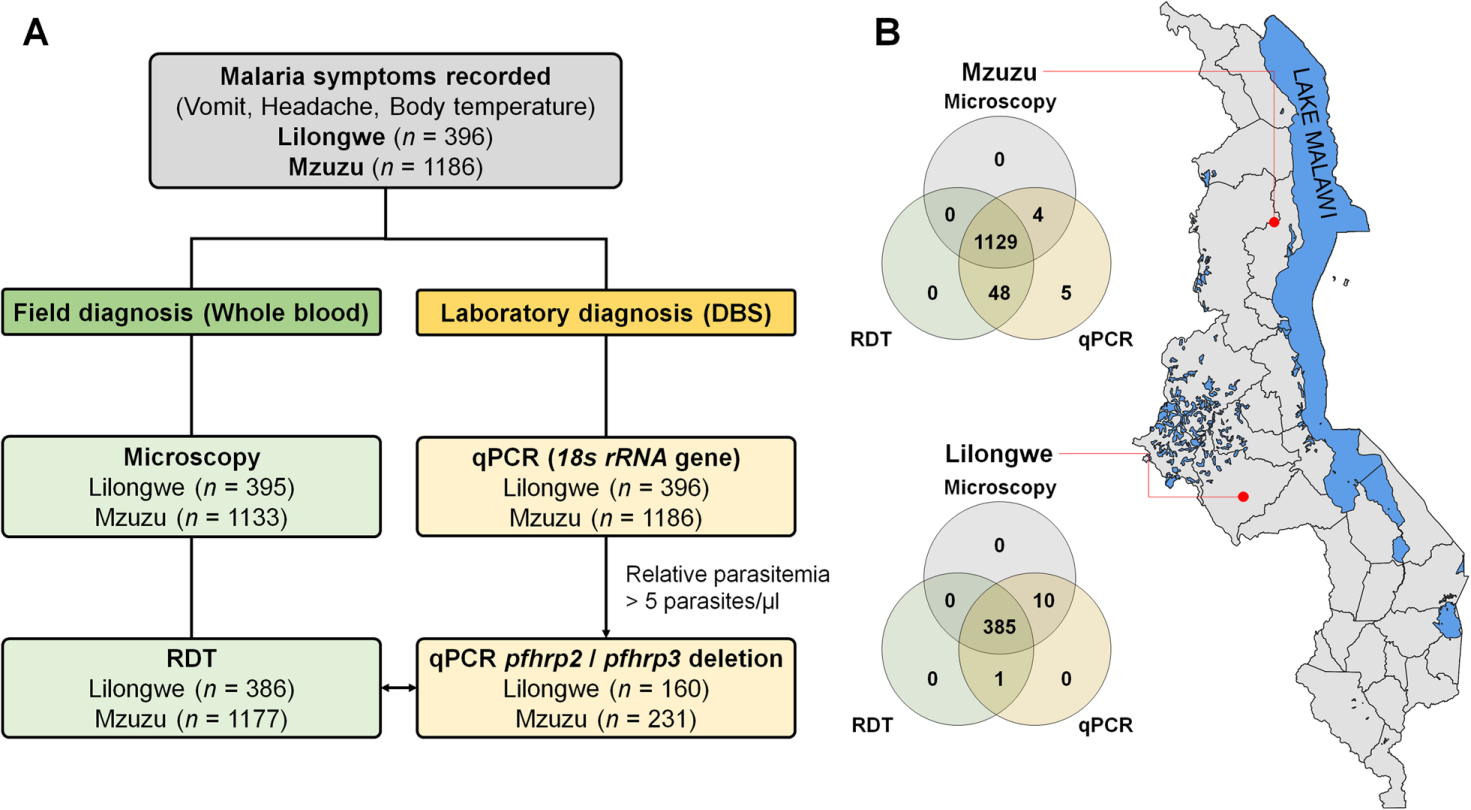
Flowchart of clinical samples processing and map of the sampling region.** A** The flowchart illustrates the number of successful diagnoses by each field diagnostic tool and region, with results shown in the green boxes. The laboratory tests performed using quantitative PCR (qPCR) for all participants are displayed in the yellow boxes. qPCR-positive samples were selected for the *pfhrp2* and *pfhrp3* gene deletion study and compared with RDT results. **B** The map shows the location of the clinical isolate collection sites, where *Plasmodium falciparum* infected participants were enrolled. Mzuzu and Lilongwe in Malawi are indicated by red circles. The connection lines in the Venn diagram represent the number of participants successfully diagnosed by each field diagnostic method which included microscopy and RDT

Samples were collected from Lilongwe and Mzuzu referral hospitals in Malawi from December 2020 to June 2021. The study targeted individuals of all age groups who were visiting the hospitals for treatment. The two referral hospitals were purposively selected because they serve a large proportion of the population and are in high malaria transmission settings. Approximately 15% of Malawi’s population resides in Lilongwe, while Mzuzu is reported to be the fastest-growing city in terms of population. Both regions are reported to have among the highest malaria risk populations [18]. Prior to participation, written informed consent was obtained from all adult participants. For participants under 18 years of age, assents were obtained from the minors, and written informed consent was provided by their parents or legal guardians. All consent and assent procedures were conducted in accordance with the approved study protocol and ethical guidelines. In this study, a total of 1582 patient blood samples were collected, 396 (25.0%) blood samples from Lilongwe and 1186 (75.0%) blood samples from Mzuzu hospitals (Fig. 1A and B). The sample size was not statistically predetermined; instead, it was based on the number of eligible participants available during the study period. This convenience sampling approach aimed to collect as many samples as possible to support meaningful molecular and diagnostic analyses.

### Clinical sample collection and handling

All procedures were performed by trained technicians at local hospitals. At each selected site, participants were screened for eligibility, and 100 µl of finger-prick blood was collected after obtaining voluntary informed consent. Two Giemsa-stained thin smears were prepared from two drops of blood for microscopic diagnosis. Each slide was independently examined by two microscopists, who were blinded to each other’s results. If both identified malaria parasites of the same species, the sample was confirmed as positive. In cases of disagreement, a third microscopist blinded to the earlier results, re-examined the slide to make the final decision. All microscopists received formal training, underwent competency assessments, and participated in regular internal quality control and external quality assurance (EQA) programs to ensure diagnostic accuracy.

An additional 10 µl of blood was applied to a *Pf*HRP2-RDT (Paracheck-Pf® version 3, Orchid Biomedical Systems, Goa, India; Catalog number: 302030025) as part of the routine malaria diagnostic procedure in local health facilities. A few drops of blood were also spotted onto Whatman filter paper to prepare dried blood spots (DBS). DBS papers were individually packed in plastic bags with desiccants and shipped to Kangwon National University (KNU) in the Republic of Korea, where qPCR was performed to confirm malaria diagnosis and detect *pfhrp2/3* gene deletions.

### *P. falciparum *in vitro culture and gDNA extraction for *pfhrp2/3* control preparation

To determine the limit of detection (LoD) of qPCR diagnosis targeting the *18S rRNA* gene and *pfhrp2/3* detection based on parasite count, *P. falciparum* strains 3D7 (ATCC) (wildtype, west Africa origin), Dd2 (*pfhrp2* deletion, Indochina origin), and HB3 (*pfhrp3* deletion, Honduras origin) were cultured using human erythrocytes at 2% *hematocrit* in RPMI-1640 medium (Gibco: Thermo Fisher Scientific, Inc., Waltham, MA, USA). The culture medium was supplemented with 2.3 g/L sodium bicarbonate, 0.05 g/L hypoxanthine, 10% Albumax I solution, and 10 mg/ml gentamicin. Cultures were maintained in a gas mixture of 90% N_2_, 5% O_2_, and 5% CO_2_ and incubated at 37 °C. Thin blood films were prepared following standard protocols, and parasitemia was measured by Giemsa staining under a microscope at 100 × magnification. Once the ring-stage of parasites reached 5% after synchronization, genomic DNA (gDNA) was extracted from the cultured samples [19]. For the clinical isolates gDNA extraction, one spot (10 mm in diameter, approximately 50 μl) was punched from each dried blood spot (DBS) paper, cut into several pieces, and transferred into individual sterile 1.5 ml microcentrifuge tubes. DNA was extracted using the QIAamp DNA Mini Kits (Qiagen, Hilden, Germany), according to manufacturer’s instructions with 50 μl of final elution.

### Molecular diagnosis of *P. falciparum* infections by quantitative PCR (qPCR)

Molecular diagnosis targeting the *18S rRNA* gene of* P. falciparum* was performed using qPCR with previously published primers (Forward primer: ATTGCTTTTGAGAGGTTTTGTTACTTT, Reverse primer: GCTGTAGTATTCAAACACAATGAACTCAA) and a probe (FAM-CATAACAGACGGGTAGTCAT) [20]. The qPCR assay components and cyclic conditions were slightly modified to optimize performance. Briefly, each qPCR reaction was conducted in a total volume of 20 µl, including 10 µl of 2 × Prime Time Gene expression Master Mix with ROX reference dye (Integrated DNA technologies, Coralville, IA, USA), 1 µl of template DNA (gDNA), 0.5 µmol/L of each of the primers, and 0.25 µmol/L of each *Taq*Man Probe. Reactions were run on an AriaMx Real-Time PCR system (Agilent Technologies, Santa Clara, CA, USA) under the following cycling conditions: an initial hot-start polymerase activation at 95 ℃ for 10 min, followed by 45 cycles of denaturation at 95 ℃ for 15 s and annealing/extension at 65 ℃ for 65 s. A standard curve for the qPCR diagnosis was prepared using *P. falciparum* 3D7 strain parasites cultured in vitro at 5% ring-stage. All qPCR reactions were performed in duplicate, and for each test, a tenfold serial dilution of the standard curve was included.

### Characterization of *pfhrp2* and *pfhrp3* deletions in *P. falciparum* positive samples

A multiplex qPCR was performed to identify *pfhrp2* and *pfhrp3* deletions. All reactions were run parallel with genomic DNA from cultured *P. falciparum* 3D7 as a standard control. Published primer sequences for *pfhrp2* (Forward: GTATTATCCGCTGCCGTTTTTGCC; Reverse: CATCTACATGTGCTTGATTTTCGT) and *pfhrp3* (Forward: ATATTATCCGCTGCCGTTTTTGCT; Reverse: CCTGCATGTGCTTGACTTTCGT) and probes (*pfhrp2*: FAM-TTCCGCATTTAATAATAACTTGTGTAGC, and *pfhrp3*: HEX-CTCCGAATTTAACAATAACTTGTTTAGC) were adapted for the multiplex qPCR platform [21].

These primers and probes targeting *pfhrp2* (PF3D7_0831800) and *pfhrp3* (PF3D7_1372200) were designed to amplify a region that includes exon 1, the intron, and the initial 96 base pairs of exon 2 (Supplementary Table 1). This specific region was selected based on previous studies highlighting its usefulness in detecting gene deletions associated with RDT failure in field samples. A lack of amplification in this region was considered indicative of either complete or partial gene deletion. Although the antigenic epitopes recognized by HRP2-based RDTs are located further downstream within exon 2, the targeted upstream region has been shown to reliably reflect diagnostically significant deletions [22, 23]. These primers were tested with varying gDNA concentrations extracted from *Pf3D7*, *PfDd2,* and *PfHB3* strains, corresponding to parasite counts in tenfold serial dilution from 210,000 parasites/µl to 0.21 parasites/µl. Reactions were conducted in a total volume of 10 µl, including 5 µl of 2 × Prime Time Gene expression Master Mix with ROX reference dye (Integrated DNA technologies), 1 µl of template DNA (gDNA), 0.5 µmol/L of each of the primers, and 0.25 µmol/L of each *Taq*Man Probe. Reactions were conducted on an AriaMx Real-Time PCR system (Agilent Technologies) with the following cycling conditions: hot-start polymerase activation at 95 ℃ for 5 min, followed by 45 cycles of denaturation at 95 ℃ for 15 s and annealing/extension at 60 ℃ for 35 s.

To establish molecular surveillance criteria for the *pfhrp2* and *pfhrp3* genes, a cutoff of relative parasitemia based on the *18S rRNA* gene of under 5 parasites/µl, as described elsewhere, was implemented [24]. Hence, samples with less than 5 parasites/µl in *18S rRNA* gene detection were considered analytically negative for *P. falciparum* infection and were excluded from *pfhrp2* and *pfhrp3* gene detection. A Cq value of ≤ 35 was considered positive, while samples with Cq > 35 were interpreted as negative. No template controls (NTCs) containing nuclease-free water were included in each run to monitor contamination.

### Participant inclusion and case definitions

This study enrolled both symptomatic and asymptomatic individuals who were diagnosed with malaria. There were no exclusion criteria based on disease severity or symptom presentation; all malaria-positive cases identified during the study period were included.

Symptomatic malaria is defined by the presence of malaria-related symptoms (vomiting, headache, and body temperature > 37 °C) within the past 2 days and at the time of examination, along with detectable malaria parasites in blood. Clinical manifestations data were collected using a structured questionnaire administered to all participants, which included questions about the presence of these symptoms within the specified timeframe. Asymptomatic malaria is defined as the presence of detectable parasites in the blood without any malaria-related symptoms during the past week or at the time of the survey, and with no history of antimalaria drug use within the past week.

### Diagnostic result definitions

In this study, false negative results were defined as samples that tested negative by both rapid diagnostic test (RDT) and quantitative PCR (qPCR) but were positive by light microscopy (LM). True positive results were defined as samples that tested positive by both RDT and qPCR.

### Statistical analysis

The qPCR data were visualized by Agilent AriaMx 1.8 software (Agilent Technologies) and statistically analyzed with GraphPad Prism version 8.0.2 (GraphPad software, Inc., San Diego, CA, USA). A log transformation of parasitemia was performed to compare detection by microscopy and qPCR. Correlation analysis of parasitemia was conducted using Spearman's correlation (*ρ*) and linear regression via Sigma Plot software v12 (Systat Software, Inc., San Jose, CA, USA). Actual parasitemia measured by microscopy and relative parasitemia from *18S rRNA* qPCR were summarized as means and correlated to assess the agreement between log-transformed parasite values. An unpaired *t*-test was used to compare the two diagnostic methods, while differences in asymptomatic and symptomatic cases between the two regions were analyzed using the Mann–Whitney test. The Wilcoxon signed-rank test was used to compare paired numeric values. Statistical analysis of clinical specimen diagnosis was conducted with MedCalc statistical, available at https://www.medcalc.org/calc/diagnostic_test.php. Data were organized into double-entry tables to calculate sensitivity, specificity, and positive and negative predictive values for each test at 95% confidence intervals.

### Ethics statement

The study procedures were approved by the National Health Science Committee (NHSRC), a division of Malawi’s Ministry of Health (MoH) (IRB00003905—Evaluation of Diagnostic Accuracy of the Next Generation Mobile Malaria Diagnostic Kit (miLab™)), as well as by the Ethical Review Board at Kangwon National University (KWNUIRB-2023-05-008). Before participating in the study, all participants provided informed consent. All experiments were conducted in accordance with relevant guidelines and regulations.

## Results

### Prevalence of *P. falciparum* in Lilongwe and Mzuzu, Malawi

A total of 1582 participants were enrolled in the study, including 396 (25.0%) from Lilongwe and 1186 (75.0%) from Mzuzu (Fig. 1A and B). Malaria-related symptoms were recorded for all participants at the time of sample collection. Of the samples collected, 1528 (96.6%) and 1563 (98.8%) were successfully tested using light microscopy (LM) and malaria rapid diagnostic tests (RDT), respectively (Fig. 1A and B). A total of 1514 (95.7%) samples were tested by both diagnostic methods. Molecular diagnosis for *P. falciparum* was performed on all 1582 (100%) isolates using qPCR (Fig. 1A and B).

Overall, malaria positive rate based on field-based diagnostic tools was significantly higher in Lilongwe (30.6% by LM and 53.4% by RDT) compared to Mzuzu (21.0% by LM and 21.2% by RDT). Across both regions, the overall prevalence was 23.5% by LM and 29.2% by RDT (Table 1). In contrast, the laboratory-based qPCR method detected a higher overall prevalence of 31.4% (497/1582 samples) compared to the field diagnostic tools (Table 1).

**Table 1:** *Plasmodium falciparum* prevalence based on each diagnostic tools in Lilongwe and Mzuzu, Malawi

Diagnostic tool	Lilongwe			Mzuzu			Both Sites		
	No. of positive (%)	No. of negative (%)	Total (%)*	No. of positive (%)	No. of negative (%)	Total (%)*	No. of positive (%)	No. of negative (%)	Total (%)*
Microscopy	121 (30.6)	274 (69.4)	395 (99.7)	238 (21.0)	895 (79.0)	1133 (95.5)	359 (23.5)	1169 (76.5)	1528 (96.6)
RDT	206 (53.4)	180 (46.6)	386 (97.5)	250 (21.2)	927 (78.8)	1177 (99.2)	456 (29.2)	1107 (70.8)	1563 (98.8)
qPCR	214 (54.0)	182 (46.0)	396 (100.0)	283 (23.9)	903 (76.1)	1186 (100.0)	497 (31.4)	1,085 (68.6)	1582 (100.0)

*RDT* Rapid Diagnostic Test, *qPCR* quantitative Polymerase chain reaction, % percentage

^*^Total number of isolates accessible by each diagnostic tool at each site

### Comparison of field diagnostic performance and the effect of parasite density

**Fig. 2:**
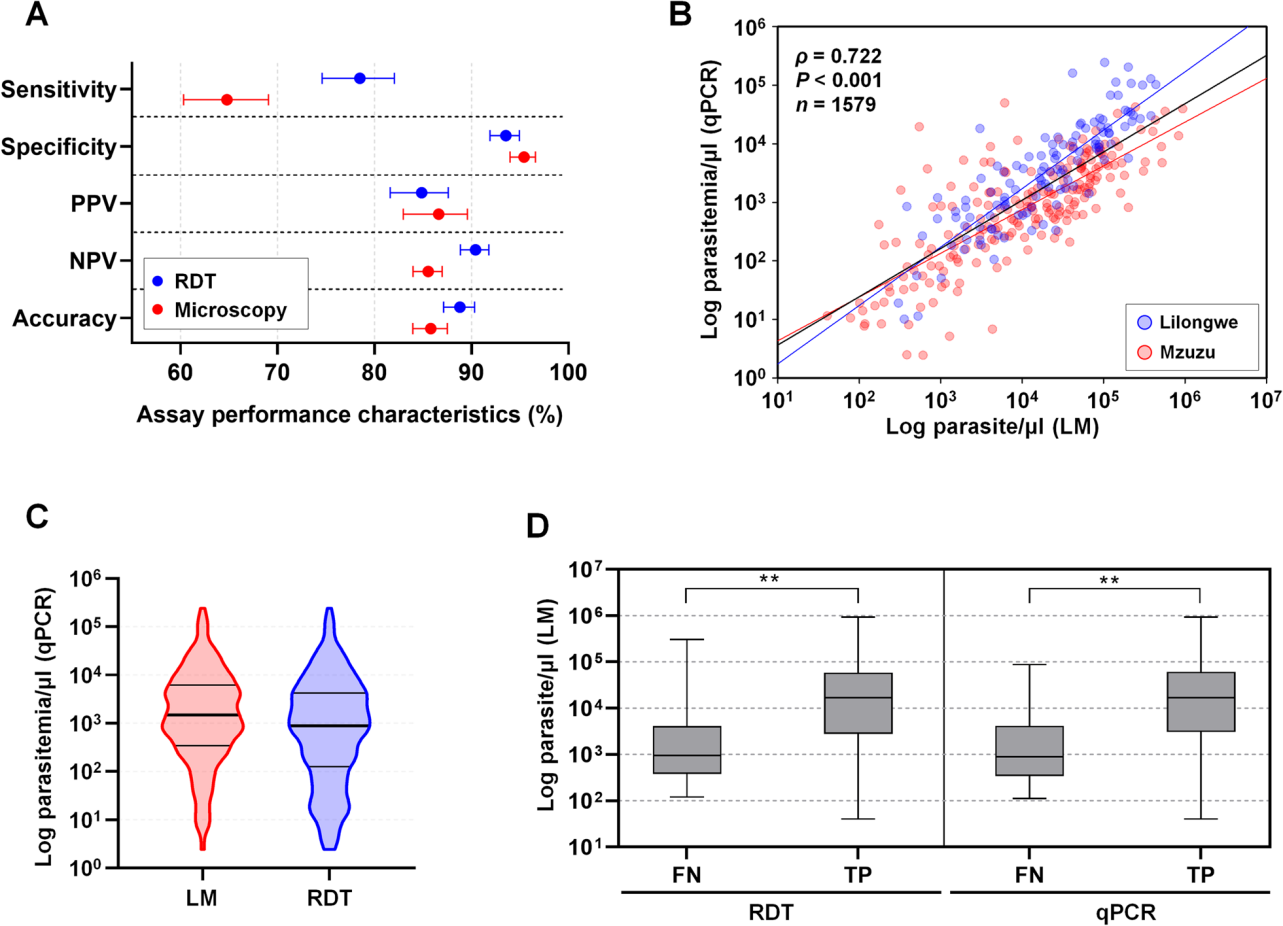
Analytical validation of field diagnosis performance compared with laboratory tests. **A** Analytical validation of the performance of each field diagnostic tool was assessed by comparing it to quantitative PCR. Standard parameters such as sensitivity, specificity, positive predictive value (PPV), negative predictive value (NPV), and accuracy including their 95% confidence intervals, are presented. **B** The Spearman's correlation between actual parasitemia under light microscopy (LM) and parasite density determined using qPCR was calculated using cultured *P. falciparum* 3D7 strain gDNA. The parasitemia was converted to parasites per microliter to compare between field and laboratory tests. The black line indicates the combined data from both study sites (Lilongwe and Mzuzu), with a total of 1579 isolates used for comparison, showing a significant positive correlation (*ρ* = 0.722). **C** The violin plot represents the distribution of qPCR-based relative parasitemia for LM and RDT positive groups. The three horizontal lines within each violin indicate the interquartile range and median. Significance was assessed by a paired *t*-test (*P* = 0.034). The average and min-to-max range are shown in the violin plot for each field diagnosis compared to relative parasitemia calculated by qPCR. **D** Actual parasitemia detected by LM is compared for each diagnostic tool, showing false negative (FN) or true positive (TP) results. The two stars (**) indicate significant differences between the groups. Statistical comparison was performed using the Wilcoxon signed-rank test. The difference in parasitemia between FN and TP was significant for both methods: RDT (*P* = 0.0031, **) and qPCR (*P* = 0.0054, **). The number of false negatives (FN) and true positives (TP) for each method are indicated in parentheses: RDT (FN = 40, TP = 319) and qPCR (FN = 50, TP = 311)

In reference to qPCR, RDT showed higher sensitivity of 78.5% [95% confidence interval (*CI*): 74.6–82.1%] but lower specificity at 93.6% (95% *CI*: 92.0–95.0%) when compared to light microscopy (LM), which had a sensitivity of 64.8% (95% *CI*: 60.3–69.1%) and specificity of 95.4% (95% *CI*: 94.0–96.6%) (Fig. 2A). The positive predictive value (PPV), which is the probability that the disease is present when the test is positive, was higher for microscopy (LM) at 86.6% (95% *CI:* 83.0–89.6%), while the negative predictive value (NPV), which is the probability that the disease is not present when the test is negative, was higher for RDT at 90.4% (95% *CI*: 88.9–91.8%). Diagnostic accuracy in this study was defined as the proportion of true positives and true negatives among all individuals tested and was higher for RDT (88.8%, 95% *CI*: 87.1–90.3%) than for LM (85.8%, 95% *CI*: 84.0–87.5%) (Fig. 2A).

Under LM detection, parasitemia was significantly lower in Mzuzu (Mean ± *SD*: 46,805 ± 113,152 parasites/μl) than in Lilongwe (Mean ± *SD*: 57,931 ± 85,397 parasites/μl) (*P* < 0.001). A strong positive correlation was observed between parasitemia quantified by LM in the field and relative parasite density estimated by *18S rRNA* based qPCR in the laboratory (*ρ* = 0.722, *P* < 0.001), indicating a consistent association between the two methods (Fig. 2B). However, regression analysis suggested that qPCR may systematically yield lower parasite density estimates compared to LM. The parasitemia levels detected by field diagnostic methods differed significantly, with LM showing a median of 1488 parasites/μl (IQR: 346.1–6224) and RDT showing a median of 882.4 parasites/μl (IQR: 126.8–4235) (*P* = 0.0344) (Fig. 2C). Based on parasite density, the performance of both LM and RDT was evaluated for infections above 2.4 parasites/μl, which corresponds to the detection limit of the qPCR method used as the reference standard in the study regions (Fig. 2C).

When comparing LM to RDT and qPCR, the differences in parasitemia between false negative (FN) and true positive (TP) results showed varying average parasitemia levels (Fig. 2D). False negative cases had averages of 9964.5 parasites/μl in RDT, and 8961.4 parasites/μl in qPCR, while true positive cases exhibited averages of 55,431.0 parasites/μl in RDT, and 56,918.6 parasites/μl in qPCR (Fig. 2D). These differences in parasitemia levels between FN and TP detected by RDT (*P* = 0.0031) and qPCR (*P* = 0.0054), when compared with LM, were statistically significant (Fig. 2D). These results indicate that the sensitivity of RDT and qPCR is strongly influenced by parasite density.

### Distribution of parasitemia levels and associated symptoms in malaria positive participants

**Fig. 3:**
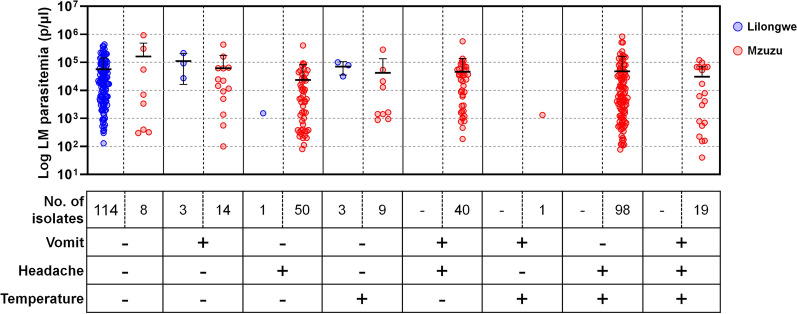
Distribution and association of parasitemia by LM (parasites/μl) and clinical manifestations. Each symptom presentation is indicated by “ + ” (present) and “−” (absent). Parasitemia measured by LM was compared between clinically recorded isolates from Lilongwe and Mzuzu, revealing statistically significant differences between the two regions (Mann-Whitney test, *P* < 0.001)

Participants were recruited based on their availability at local hospitals, rather than clinical suspicion of malaria. To assess differences in clinical manifestation patterns across the study regions, symptoms related to malaria, including vomiting, headache, and body temperature (> 37 °C), were recorded. In Lilongwe, 114 out of 121 (94.2%) *P. falciparum*-positive participants identified by LM did not have any specific symptoms related to malaria, while the remaining 7 (5.8%) were symptomatic (Fig. 3). In contrast, in Mzuzu, 231 out of 238 (97.1%) LM positive participants were symptomatic, with either single or mixed clinical manifestation, while 8 participants (3.4%) were asymptomatic (Fig. 3). Among the asymptomatic individuals from both Lilongwe and Mzuzu, LM positive isolates did not show a statistically significant difference (*P* = 0.43). The overall average parasitemia under LM was significantly different between clinically recorded isolates in Lilongwe (*n* = 121, 57,931 ± 85,397 parasites/μl) and Mzuzu (*n* = 239, 45,262 ± 111,561 parasites/μl) (*P* < 0.0002). These results clearly indicate that the degree of symptom onset differed significantly between the two regions, while parasitemia levels did not differ.

### Molecular validation of *pfhrp2* and *pfhrp3* gene deletions using qPCR in field isolates

**Fig. 4:**
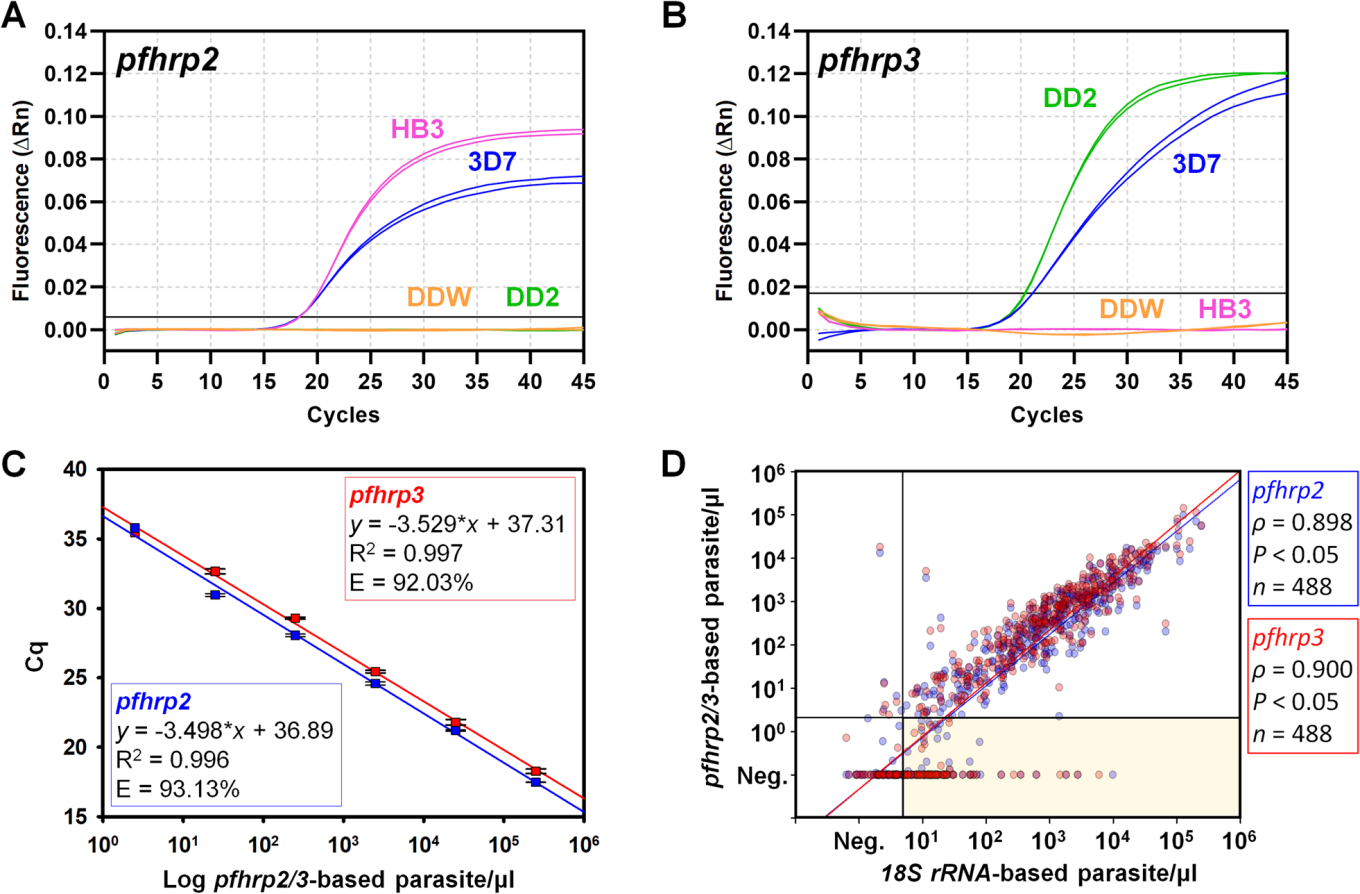
Validation of multiplex qPCR assay for *pfhrp2* and *pfhrp3* gene deletion detection. **A** Laboratory *P. falciparum* strains 3D7 (wild type, *pfhrp2* and *pfhrp3* present), Dd2 (*pfhrp2* deletion), and HB3 (*pfhrp3* deletion) were amplified under optimal multiplex qPCR conditions. The black horizontal line marks the threshold for *pfhrp2* analysis and **B** shows multiplex qPCR for *pfhrp3* gene. **C** A standard curve of *pfhrp2* and *pfhrp3* gene amplification using tenfold serially diluted parasites, starting from 210,000 parasites/μl. The assay was able to detect as low as 2.1 parasites/μl with an amplification efficiency of 93.13% for *pfhrp2* and 92.03% for *pfhrp3*. The regression slope was − 3.498 for *pfhrp2* and − 3.529 for *pfhrp3* gene amplification. The coefficient of determination (R^2^) for both targets was above 0.99, indicating a strong linear correlation. **D** A direct comparison between the ratio of *pfhrp2/3* genes to *18S rRNA* gene quantity is shown. The double negative population in the fourth quadrant is considered to have fewer than 2.1 parasites/μl for *pfhrp2/3* genes and fewer than 5 parasites/μl for the *18S rRNA* gene. The yellow box indicates *18S rRNA-*positive isolates with undetectable *pfhrp2* or *pfhrp3* gene amplification, indicating possible gene deletions

To detect and confirm molecular surveillance of *pfhrp2* and *pfhrp3* gene deletions, a multiplex qPCR-based assay was performed. Genomic DNA from three different *P. falciparum *in vitro cultured strains (3D7, Dd2, and HB3) were used to optimize and validate the assay for *pfhrp2* and *pfhrp3* gene deletion status. The *P. falciparum* 3D7 strain contains both the *pfhrp2* and *pfhrp3* genes, while the Dd2 strain has a single gene deletion of *pfhrp2,* and the HB3 strain has a deletion of the *pfhrp3* gene (Fig. 4A and B). To establish molecular surveillance criteria for detecting deletion of *pfhrp2* and *pfhrp3* genes in clinical isolates, a cutoff of relative parasitemia below 5 parasites/μl based on the *18S rRNA* gene was employed as described elsewhere [24]. Relative parasitemia in *pfhrp2-* and *pfhrp3-*deleted isolates was estimated by comparing the Cq values of these target genes to those of a reference gene (*P. falciparum* 3D7 strain). This relative quantification approach was used to determine the presence or absence of *pfhrp2* and *pfhrp3* gene targets based on their amplification profiles.

The analytical limit of detection (LoD) for the multiplex qPCR targeting both *pfhrp2* and *pfhrp3* genes using the *P. falciparum* 3D7 strain was estimated to be 2.1 parasites/μl, with qPCR efficiencies of 93.1% and 92.3%, respectively (Fig. 4C). The LoD for the *pfhrp2* and *pfhrp3* genes was approximately 10 times higher than that of the *18S rRNA* gene (0.21 parasites/μl) when detected by qPCR. A significant positive correlation was observed between the *18S rRNA* gene and *pfhrp2* (*ρ* = 0.898), and between *18S rRNA* and *pfhrp3* (*ρ* = 0.900) (Fig. 4D). In this analysis, samples with less than 5 parasites/μl based on *18S rRNA* gene detection were considered analytically negative and excluded from further gene deletion analysis (Fig. 4D).

### Deletions of *pfhrp2 and pfhrp3* genes in isolates from Mzuzu and Lilongwe, Malawi

Multiplex qPCR assays were performed on 488 clinical isolates confirmed as *P. falciparum* positive by qPCR to assess *pfhrp2* and *pfhrp3* gene deletion. Based on the inclusion criteria for gene deletion analysis, 160 isolates from Lilongwe and 231 isolates from Mzuzu were included in the final dataset. In Lilongwe, *pfhrp2* single gene deletions were detected in 7 isolates (4.4%) with a relative parasite density of 1460 ± 3705 parasites/μl, calculated from the *18S rRNA* gene (Table 2). In Mzuzu, 2 isolates (0.9%) showed *pfhrp2* single gene deletions, with a relative parasite density of 11.05 ± 0.70 parasites/μl, which was marginally lower than in Lilongwe. Single *pfhrp3* gene deletions were detected in 11 isolates (6.9%) in Lilongwe and 11 isolates (4.8%) in Mzuzu. The parasite density was 615.1 ± 1785 parasites/μl in Lilongwe and 168.5 ± 433.4 parasites/μl in Mzuzu (Table 2). Dual gene deletions, which lead to false negatives in RDT, were detected in 24 isolates (15.0%) in Lilongwe and 24 isolates (10.4%) in Mzuzu, with parasite densities of 26.36 ± 37.52 parasites/μl and 167.2 ± 570.5 parasites/μl, respectively (Table 2). Overall, deletions in either the *pfhrp2*, *pfhrp3* or both genes were confirmed in 79 isolates (20.2%) in Malawi (Table 2).

**Table 2:** *pfhrp2* and *pfhrp3* gene deletion status in *P. falciparum* isolates from Lilongwe and Mzuzu, Malawi

	*Δpfhrp2*		*Δpfhrp3*		*Δpfhrp2* and *3*		No deletion		Total
	*n* (%)	RPD	*n* (%)	RPD	*n* (%)	RPD	*n* (%)	RPD	*n*
Lilongwe	7 (4.4)	1460 ± 3705	11 (6.9)	615.1 ± 1785	24 (15.0)	26.36 ± 37.52	118 (73.8)	17,291 ± 38,647	160
Mzuzu	2 (0.9)	11.05 ± 0.70	11 (4.8)	168.5 ± 433.4	24 (10.4)	167.2 ± 570.5	194 (84.0)	3408 ± 7039	231
Total	9 (2.3)		22 (5.6)		48 (12.3)		312 (79.8)		391

^*^RPD relative parasite density (parasites/μl, Mean ± standard deviation), *n* number of isolates*, %* percentage

## Discussion

This study was conducted to determine the prevalence of *P. falciparum* in Malawi and the contribution of *pfhrp2* and *pfhrp3* gene deletion to the effectiveness of malaria diagnostic tools. As the Global Technical Strategy on Malaria 2016 − 2030 aims for a malaria-free world by 2030, surveillance of *pfhrp2/3* deletions is essential for monitoring progress towards this goal [25]. As malaria transmission declines in endemic countries, the proportion of low-density infections among both symptomatic and asymptomatic individuals is likely to increase, which may reduce the utility of light microscopy and malaria RDTs. Both methods have been shown to underestimate malaria prevalence in such cases.

In Malawi, as in many malaria-endemic countries, LM and RDTs remain the primary tools for malaria diagnosis. Therefore, it is essential to evaluate the effectiveness of these diagnostic tools. Molecular techniques, particularly PCR-based methods, are known for their higher sensitivity in malaria detection [26–28]. Our findings indicate that while both LM and RDTs are highly specific, LM generally has a lower detection rate compared to RDTs [29, 30]. This is due to several factors that limit LM efficiency, including reliance on reader expertise, slide preparation quality, and its detection threshold. Although HRP2/3-based RDTs offer higher sensitivity, they can produce false-positive results due to the prolonged persistence of HRP2/3 antigens following parasite clearance [31, 32]. In this study, when qPCR used as a reference standard, RDT showed a higher sensitivity (78.50%) making them more effective than LM in detecting malaria in the studied regions. Conversely, LM showed higher specificity (95.42%) compared to RDTs. This aligns with a study from Nigeria [33], suggesting that RDTs may detect low-density infections or lingering HRP2/3 antigens post-infection, which LM could miss [34]. So far, comparative analysis of LM and RDT provided the rate of false positives and true positives both of which have implications for malaria control and intervention in Malawi.

In this study, we found that the prevalence rate of asymptomatic malaria was higher in Lilongwe (28.9%) than Mzuzu district (0.8%). This finding was comparable to a study conducted from December 2019 to April 2020 on asymptomatic malaria in blood donors from the central zone of Malawi, which reported 18.5% asymptomatic malaria cases in Lilongwe and 8.6% in Mzuzu from northern zone [35]. Other studies in Malawi have reported asymptomatic malaria prevalence rates of 42% among school age children, and 14.1% in younger children [36, 37]. Similar trends have been observed in countries such as Ethiopia (Pawe, 14.5%) [38], Nigeria (77.6%) [39], Cameroon (Douala, 28.9%) [40], and Tanzania (Bagamoyo district and other regions, 57.5%) [41, 42]. These similarities are likely due to high transmission and repeated exposure, which promote the development of immunity and asymptomatic infection [43]. Differences in asymptomatic malaria prevalence rates can also be attributed to differences in geographical locations, study design, housing quality, population characteristics, sample size, study period, vector control methods, and malaria transmission rates [43]. In this study, parasite densities did not differ significantly between symptomatic and asymptomatic individuals, indicating that silent carriers may contribute substantially to malaria transmission. Thus, we recommend enhanced surveillance of both symptomatic and asymptomatic malaria in Malawi, along with an evaluation of the detection limits of primary diagnostic tools used in the region.

The recent emergence of parasites lacking *pfhrp2* and *pfhrp3* genes poses a threat to malaria diagnosis and control programmes [44]. The WHO recommends reconsidering the use of HRP2-based diagnostics when more than 5% of clinical *P. falciparum* infections produce false-negative RDT results due to *pfhrp2/3* gene deletions [17, 45]. Although *pfhrp2/3* gene deletions had not previously been reported in Malawi, our study detected *pfhrp2* deletions in 2 (0.9%) samples from Mzuzu and 7 (4.4%) samples from Lilongwe. These single gene deletions did not lead to false-negative RDT results, consistent with findings from previous studies from Tanzania and Yemen [46, 47]. This anomaly could be the consequence of a false-positive RDT result due to cross-reactivity with circulating proteins such as rheumatoid factor in the bloodstream [48], or it could be the result of a prior infection with samples that tested positive for *pfhrp2* [49]. Additionally, we observed single *pfhrp3* deletions in 4.8% of samples from Mzuzu and 6.9% from Lilongwe. These data further reveal a higher proportion of *pfhrp3* deletions compared to *pfhrp2*. This finding is significant, as *pfhrp3* deletions are believed to occur more prevalent during low transmission seasons, when polyclonal infections are less likely. Similar observations have been identified in Central and Southern America, where malaria transmission is low, with up to 70% of tested samples showing deletions in the *pfhrp3* region [50–52]. Dual deletions were detected in 48 samples (12.3%), which were not detected by RDTs, indicating false-negative results and raising significant concerns for malaria diagnosis in the region. These deletions were observed in both symptomatic and asymptomatic populations, across both low and high parasitemia. Since RDTs are the primary diagnostic tool in Malawi, false negatives due to gene deletions could lead to missed diagnoses, undermining malaria control efforts. Vigilant monitoring of *pfhrp2/3* deletions is therefore critical to avoid undetected infections that could compromise malaria eradication goals. Similar effects of dual deletions have been observed in other African regions [44].

This study has several important limitations. First, the samples used in this study were collected between 2020 and 2021; therefore, the study design did not follow the WHO protocol for assessing *pfhrp2* deletions [45]. As a result, our prevalence estimates may be overestimated or underestimated. Moreover, a low parasite density in asymptomatic cases may not be suitable for detection of *pfhrp2/3* deletions. For this reason, the WHO recommends evaluating deletions in symptomatic patients to ensure more accurate data [53]. Additionally, we did not perform genotyping (e.g., *msp1/msp2*) or assess within-sample diversity to evaluate the presence of polyclonal infections. In samples where multiple parasite clones are present, *pfhrp2/3* deletions may be masked by wild-type strains, potentially leading to an underestimation of true deletion prevalence. Despite these limitations, our use of a multiplex qPCR assay demonstrated a practical approach for detecting *pfhrp2/3* deletions even at low parasite densities [54]. Finally, as only two sites were involved in this study, the findings may not be representative of the entire country, emphasizing the need for nationwide surveillance.

## Conclusions

This study provides evidence of *pfhrp2* and *pfhrp3* gene deletions in *P. falciparum* isolates from Malawi, highlighting the urgent need for surveillance to assess their prevalence and spread across the country. The high proportion of *pfhrp3* deletions requires further investigation into the factors influencing these deletions during the transmission season. Based on these findings, we recommend screening for *pfhrp2/3* deletions even in RDT-positive samples, considering the potential for cross-reactivity and false positives caused by lingering *pfhrp2/3* antigens after treatment.

Our comparative analysis showed that, although RDTs remain widely used, their diagnostic sensitivity is compromised in areas with a high prevalence of gene deletions. In contrast, while light microscopy offered greater specificity, it lacked the sensitivity of qPCR, which emerged as the most reliable and accurate method for detecting *P. falciparum* infections. Overall, this study highlights the critical need to develop alternative diagnostic targets to address the growing challenge posed by *pfhrp2/3* deletions in malaria-endemic regions.

## Supplementary Information

Additional file 1.

## Data Availability

The datasets used and/ or analyzed during the current study are available from the corresponding author on reasonable request.

## Abbreviations

bp: Base pairs
DBS: Dried Blood Spot
EQA: External quality assurance
FN: False negative
HRP2-based RDTs: Histidine rich protein 2-based rapid diagnostic tests
IQR: Interquartile range
LDH: Lactate dehydrogenase
LM: Light microscopy
LOD: Limit of detection
NPV: Negative predictive value
*pfhrp2*: *Plasmodium falciparum histidine rich protein 2*
*pfhrp3*: *Plasmodium falciparum histidine rich protein 3*
PPV: Positive predictive value
qPCR: Quantitative polymerase chain reaction
RDTs: Rapid diagnostic tests
RPD: Relative parasite density
TP: True positive
WHO: World Health Organization
