# Supplementary material for: First report of pfhrp2 and pfhrp3 gene deletions compromising HRP2-based malaria rapid diagnostic tests in Malawi
Source: Infect Dis Poverty. 2025 Oct 9;14:98. doi: 10.1186/s40249-025-01368-8 (PMC12512526; doi:10.1186/s40249-025-01368-8)
Supplement: Supplementary file 1 — Additional file 1. [file 40249_2025_1368_MOESM1_ESM.docx]

Supplementary Table 1. Oligonucleotide sequences used in quantitative PCR assay for *pfhrp2/3* gene deletions

| Target gene | Primer / Probe name | Oligonucleotide sequence [5′ → 3′] | Amplicon size (bp) | Conc. in 5x Prime Mix (µmol/L) |
| --- | --- | --- | --- | --- |
| *Pfhrp2* gene  (PF3D7_0831800) | *Pfhrp2*_ Forward | GTA TTA TCC GCT GCC GTT TTT GCC | 288 | 1.5 |
|  | *Pfhrp2*_Reverse | TCT ACA TGT GCT TGA GTT TCG |  | 1.5 |
|  | *Pfhrp2*_Probe | 5’ 6-FAM/TTC CGC ATT/ ZEN/3’ TAA TAA TAA CTT GTG TAG C/3IABkFQ | 28 | 0.375 |
| *Pfhrp3* gene  (PF3D7_1372200) | *Pfhrp3*_Forward | ATA TTA TCC GCT GCC GTT TTT GCT | 289 | 1.5 |
|  | *Pfhrp3*_Reverse | CCT GCA TGT GCT TGA CTT TCG T |  | 1.5 |
|  | *Pfhrp3*_Probe | 5’HEX/CTC CGA ATT/ZEN/3’ TAA CAA CTT GTT TAG C/3IABkFq | 28 | 0.75 |

*All primers were premixed at a 5 ×concentration to prepare a working primer mix.

*Oligonucleotide sequences were derived from Schindler T. *et al* [21]*.*

bp base pairs, µmol/L micromoles per liter.
